# Characterization of sabatolimab, a novel immunotherapy with immuno-myeloid activity directed against TIM-3 receptor

**DOI:** 10.1093/immadv/ltac019

**Published:** 2022-08-10

**Authors:** Stephanie Schwartz, Nidhi Patel, Tyler Longmire, Pushpa Jayaraman, Xiaomo Jiang, Hongbo Lu, Lisa Baker, Janelle Velez, Radha Ramesh, Anne-Sophie Wavreille, Melanie Verneret, Hong Fan, Tiancen Hu, Fangmin Xu, John Taraszka, Marc Pelletier, Joy Miyashiro, Mikael Rinne, Glenn Dranoff, Catherine Sabatos-Peyton, Viviana Cremasco

**Affiliations:** Immuno-Oncology and Hematology, Novartis Institutes for BioMedical Research, Cambridge, MA, USA; Immuno-Oncology and Hematology, Novartis Institutes for BioMedical Research, Cambridge, MA, USA; Immuno-Oncology and Hematology, Novartis Institutes for BioMedical Research, Cambridge, MA, USA; Immuno-Oncology and Hematology, Novartis Institutes for BioMedical Research, Cambridge, MA, USA; Immuno-Oncology and Hematology, Novartis Institutes for BioMedical Research, Cambridge, MA, USA; Immuno-Oncology and Hematology, Novartis Institutes for BioMedical Research, Cambridge, MA, USA; Immuno-Oncology and Hematology, Novartis Institutes for BioMedical Research, Cambridge, MA, USA; Immuno-Oncology and Hematology, Novartis Institutes for BioMedical Research, Cambridge, MA, USA; Immuno-Oncology and Hematology, Novartis Institutes for BioMedical Research, Cambridge, MA, USA; Technical R&D GDD, Novartis Pharma Services AG., Basel, Switzerland; Technical R&D GDD, Novartis Pharma Services AG., Basel, Switzerland; Chemical Biology and Therapeutics, Novartis Institutes for BioMedical Research, Cambridge, MA, USA; Chemical Biology and Therapeutics, Novartis Institutes for BioMedical Research, Cambridge, MA, USA; Biotherapeutic and Analytical Technologies, Novartis Institutes for BioMedical Research, Cambridge, MA, USA; Biotherapeutic and Analytical Technologies, Novartis Institutes for BioMedical Research, Cambridge, MA, USA; Oncology Translational Research, Novartis Institutes for BioMedical Research, Cambridge, MA, USA; Immuno-Oncology and Hematology, Novartis Institutes for BioMedical Research, Cambridge, MA, USA; Translational Clinical Oncology, Novartis Institutes for BioMedical Research, Cambridge, MA, USA; Immuno-Oncology and Hematology, Novartis Institutes for BioMedical Research, Cambridge, MA, USA; Immuno-Oncology and Hematology, Novartis Institutes for BioMedical Research, Cambridge, MA, USA; Immuno-Oncology and Hematology, Novartis Institutes for BioMedical Research, Cambridge, MA, USA

## Abstract

**Objectives:**

Sabatolimab is a humanized monoclonal antibody (hIgG4, S228P) directed against human T-cell immunoglobulin domain and mucin domain-3 (TIM-3). Herein, we describe the development and characterization of sabatolimab.

**Methods:**

Sabatolimab was tested for binding to its target TIM-3 and blocking properties. The functional effects of sabatolimab were tested in T-cell killing and myeloid cell cytokine assays. Antibody-mediated cell phagocytosis (ADCP) by sabatolimab was also assessed.

**Results:**

Sabatolimab was shown to (i) enhance T-cell killing and inflammatory cytokine production by dendritic cells (DCs); (ii) facilitate the phagocytic uptake of TIM-3-expressing target cells; and (iii) block the interaction between TIM-3 and its ligands PtdSer/galectin-9.

**Conclusion:**

Taken together, our results support both direct anti-leukemic effects and immune-mediated modulation by sabatolimab, reinforcing the notion that sabatolimab represents a novel immunotherapy with immuno-myeloid activity, holding promise for the treatment of myeloid cell neoplasms.

## Graphical Abstract

**Graphical Abstract:**
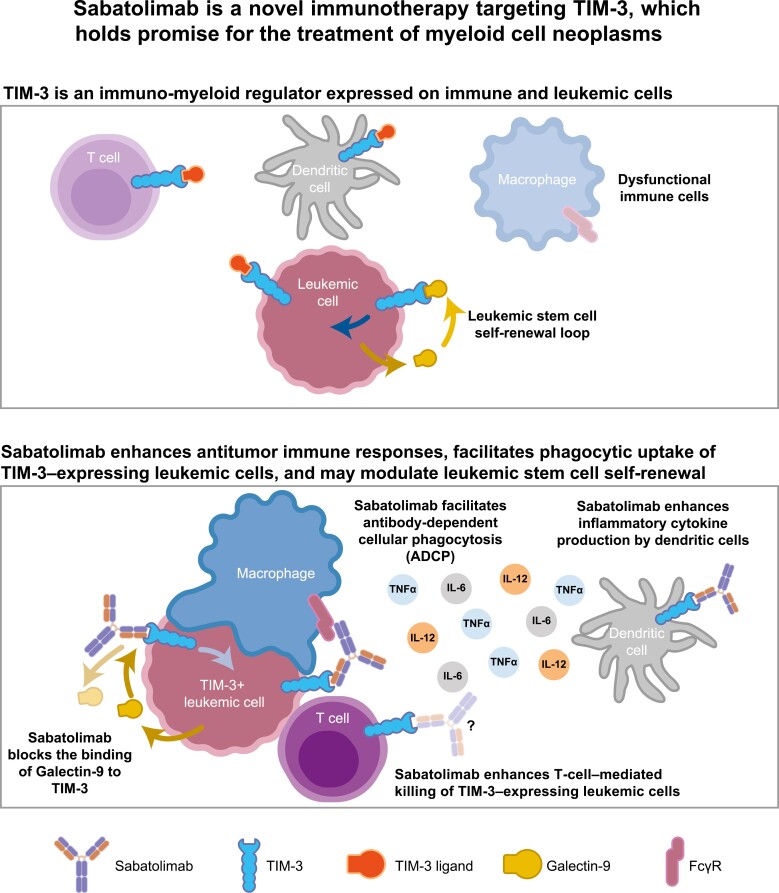

## Introduction

T-cell immunoglobulin domain and mucin domain-3 (TIM-3) is a member of the TIM family of immunoregulatory receptor proteins, for which four ligands are currently known: galectin-9, phosphatidylserine (PtdSer), high-mobility group protein B1, and carcinoembryonic antigen-related cell adhesion molecule-1 [1–5]. TIM-3 was originally discovered as a cell surface marker specific to interferon-γ producing CD4^+^ T helper 1 and CD8^+^ T cytotoxic 1 cells [6]. Although the function of TIM-3 in these cells is not fully understood, some studies have shown that its expression correlates with poor proliferation and cytokine production, thereby suggesting that TIM-3 may mark a dysfunctional population of T cells [7–10]. Notably, this is true for the T-cell exhaustion observed both in cancer and in the context of viral infections, supporting a more general association between TIM-3 and T-cell dysfunction [11]. TIM-3 expression has also been reported on the surface of regulatory T cells (Tregs), the increased presence of which correlates with disease severity in many cancer indications [12, 13]. More recently, TIM-3 has started to emerge as a broader immuno-myeloid target, in light of its expression and function on innate immune cells, including natural killer and myeloid cells. Within the myeloid cell compartment, blockade of TIM-3 on macrophages and antigen cross-presenting dendritic cells (DCs) enhances activation and inflammatory cytokine/chemokine production, ultimately leading to enhanced effector T-cell responses [2, 14–18]. In particular, loss of TIM-3 in DCs was recently associated with inflammasome activation and maintenance of CD8^+^ effector and stem-like T cells that supported anti-tumor immune responses, underscoring an important role for TIM-3 blockade in myeloid cells in the context of immunotherapies [16].

In line with the immunological properties of TIM-3, lack of cell surface expression secondary to germline mutations in HAVCR2 (the gene encoding TIM-3) results in the development of immune-related phenotypes [19–21]. In particular, these loss-of-function mutations in TIM-3 have been linked to subcutaneous panniculitis-like T-cell lymphoma, a rare cutaneous T-cell lymphoma often associated with the aberrant immunological activation of hemophagocytic lymphohistiocytosis. These patients present with increased levels of activated CD8^+^ T cells (and associated serum cytokines), as well as enhanced myeloid cell activation (increased IL-18 in serum, increased TNF-α and IL-1β upon *ex vivo* stimulation), solidifying the key role of TIM-3 as a negative regulator of both adaptive and innate immune responses [20]. Due to its critical function in inhibiting adaptive and innate immune responses, TIM-3 has been investigated in the context of anti-tumor immunity [22], and studies in animal models of cancer have shown that disruption of TIM-3 leads to a potentiation of endogenous anti-tumor immune responses [8, 10, 23].

In addition to its widespread and complex role in immune cells, TIM-3 has also been identified as a stem cell antigen in myeloid cell leukemias, with expression on the majority of CD34^+^ CD38^-^ leukemic stem cells (LSCs) and CD34^+^ CD38^+^ leukemic progenitors in acute myeloid leukemia (AML), but not on CD34^+^ CD38^-^ normal hematopoietic stem cells [24, 25]. Functional evidence for a key role for TIM-3 in AML was established by use of a TIM-3 antibody that inhibited engraftment and development of human AML in immune-deficient murine hosts [25]. In this context, TIM-3 was reported to promote an autocrine stimulatory loop via the TIM-3/galectin-9 interaction supporting LSC self-renewal [26]. Furthermore, recent clinical evidence indicated that TIM-3^+^ LSCs represent the functional clones responsible for relapse in AML patients after hematopoietic stem cell transplantation. Upregulation of TIM-3 is also associated with leukemic transformation of pre-leukemic diseases, including myelodysplastic syndromes (MDS) and myeloproliferative neoplasms, such as chronic myelogenous leukemia [27], and TIM-3 expression on MDS blasts has also been found to correlate with disease progression[28]. Altogether, these findings support the relevance of TIM-3 as an immuno-myeloid target in myeloid cell malignancies, broadening the implications for TIM-3 blockade beyond those of canonical checkpoint inhibitors such as anti-PD-1/PD-L1 agents.

Sabatolimab (also referred to as MBG453) is a high-affinity, ligand-blocking, humanized anti-TIM-3 IgG4 (S228P) antibody currently in clinical evaluation for patients with myeloproliferative disorders, including AML and MDS. Herein, we describe the development and characterization of sabatolimab, in support of its clinical development and use.

## Materials and methods

### Generation of sabatolimab

Antibodies against human TIM-3 were generated by immunizing TIM-3-deficient mice with human TIM-3-Ig fusion protein and using hybridoma technology; ELISA and flow cytometric analysis using TIM-3 transfectants (CHO, HEK, and 300.19 cells) were used to screen culture supernatant for reactivity to human TIM-3-Ig. The lead candidate monoclonal antibody, 22C1, was selected based on several factors, including binding affinity to human TIM-3 and ability to block TIM-3 interaction with one of its reported ligands, PtdSer. The murine monoclonal antibody 22C1 was then humanized, with four clones initially made and evaluated. Subsequent mutations and alternative frameworks were introduced. Ten selected humanized anti-TIM-3 clones were transiently produced in CHO cells and, among those, sabatolimab (also referred to as MBG453) was selected based on affinity for human and cynomolgus monkey TIM-3 and developability characteristics.

### Sabatolimab sequence

Heavy chain: QVQLVQSGAEVKKPGSSVKVSCKASGYTFTSYNMHWVRQAPGQGLEWMGDIYPGNGDTSYNQKFKGRVTITADKSTSTVYMELSSLRSEDTAVYYCARVGGAFPMDYWGQGTTVTVSSASTKGPSVFPLAPCSRSTSESTAALGCLVKDYFPEPVTVSWNSGALTSGVHTFPAVLQSSGLYSLSSVVTVPSSSLGTKTYTCNVDHKPSNTKVDKRVESKYGPPCPPCPAPEFLGGPSVFLFPPKPKDTLMISRTPEVTCVVVDVSQEDPEVQFNWYVDGVEVHNAKTKPREEQFNSTYRVVSVLTVLHQDWLNGKEYKCKVSNKGLPSSIEKTISKAKGQPREPQVYTLPPSQEEMTKNQVSLTCLVKGFYPSDIAVEWESNGQPENNYKTTPPVLDSDGSFFLYSRLTVDKSRWQEGNVFSCSVMHEALHNHYTQKSLSLSLG.

Light chain: AIQLTQSPSSLSASVGDRVTITCRASESVEYYGTSLMQWYQQKPGKAPKLLIYAASNVESGVPSRFSGSGSGTDFTLTISSLQPEDFATYFCQQSRKDPSTFGGGTKVEIKRTVAAPSVFIFPPSDEQLKSGTASVVCLLNNFYPREAKVQWKVDNALQSGNSQESVTEQDSKDSTYSLSSTLTLSKADYEKHKVYACEVTHQGLSSPVTKSFNRGEC

#### Cell lines and culturing conditions

The THP-1 human leukemic cell line, originally isolated by Tsuchiya and colleagues [29], was obtained from ATCC, expanded, and viably frozen at NIBR, Cambridge. The Raji human lymphoblast-like cell line, established from a Burkitt’s lymphoma of the left maxilla of an 11-year-old black male [30], was obtained from Deutsche Sammlung von Mikrooganismen Zellkulturen (DSMZ, Braunschweig, Germany) and engineered to stably express the human TIM-3 at Trenzyme (Konstanz, Germany). The SKM-1 human leukemic cell line was established from the peripheral blood of a 76-year-old Japanese man with acute monoblastic leukemia following MDS [31]. The HNT-34 human leukemia cell line was established from the peripheral blood of a 47-year-old female patient with AML secondary to previous myelodysplastic syndromes (specifically chronic myelomonocytic leukemia) [32]. Both SKM-1 and HNT-34 cells were obtained from CLEO facility within Novartis. All cell lines were thawed and cultured in RPMI-1640 medium containing 10% fetal bovine serum (FBS) for 1 week prior to use.

#### Kinetic binding studies by Biacore

A human Fab capture antibody (GE Healthcare Life Sciences) was immobilized on a CM5 chip (GE Healthcare Life Sciences) by direct amine coupling to capture sabatolimab to the CM5 chip. Sabatolimab (100 μg/ml) was captured by the human Fab antibody onto the chip at a capture level of ~50RU. Serial dilutions of human or murine TIM-3/His (Sino Biological Inc.) were used as analytes for the kinetic study. Fusion protein samples were prepared with a starting concentration of 90 nM and then diluted 1:3 in HBS-EP^+^ buffer for a total of seven concentrations. Data analysis was performed with T100 BIAevaluation software using a 1:1 binding model double reference subtraction (reference cell subtracted and blank subtracted).

#### Sabatolimab binding to 300.19 cells overexpressing TIM-3

300.19 cells engineered to express human or cynomolgus monkey TIM-3 were obtained from the lab of Gordon Freeman (Dana Farber Cancer Institute). Cells were incubated with titrated doses of sabatolimab at 4°C for 4 hours and then washed. Bound antibodies were detected with phycoerythrin (PE)-conjugated goat anti-hIgG secondary antibody (Jackson Immuno Research), and analyzed by flow cytometry. *K*_d_ values were calculated from best-fit binding curves using GraphPad Prism software.

#### TIM-3/PtdSer interaction and blocking assays

Apoptosis was induced in U937 myeloid cells (ATCC CRL-1593.2) by overnight treatment with 5 μM staurosporine (Sigma). Induction of apoptosis and exposure of PtdSer was evaluated by flow cytometric analysis after staining with Fixable Viability Dye e780 (eBioscience) and Annexin V (eBioscience). To establish a *K*_d_ for binding of TIM-3 and related TIM family member TIM-4 (both reported to bind PtdSer [3]) to PtdSer, apoptotic U937 cells were plated at 1 × 10^5^ cells/well in 96-well round bottom plates. Cells were incubated with a series dilution (0.01–100 μg/ml) of human TIM-3-Ig (R&D Systems), human TIM-4-Ig (Adipogen), or isotype control human IgG1 (R&D Systems) fusion proteins for 1 hour at 4°C in flow cytometry Buffer (phosphate buffered saline [PBS]+2% FBS) containing 2 mM CaCl_2_, followed by fluorescein isothiocyanate (FITC)-conjugated anti-human Ig secondary antibody (Sigma) for 1 hour at 4°C to reveal binding. To determine the ability of sabatolimab to block binding of TIM-3 to PtdSer, apoptotic U937 cells were incubated with solutions that contained a constant concentration of human TIM-3-Ig or human TIM-4-Ig (5 μg/ml) and serial dilutions of sabatolimab at 4°C for 1 hour. Bound human TIM-3-Ig or human TIM-4-Ig was quantified using a FITC-conjugated anti-human Ig secondary antibody, as assessed by flow cytometry. IC50 values were derived from one site—Fit logIC50 binding curves generated with Prism GraphPad software.

#### Galectin-9 competition MesoScale discovery platform (MSD) assay

Human recombinant galectin-9 was conjugated with MSD GOLD™ SULFO-TAG NHS-Ester (Mesoscale). SULFO-TAG galectin-9 was quantified using the Pierce™ Rapid Gold BCA Protein Assay Kit (Thermo Scientific). QC of SULFO-TAG-galectin-9 conjugation was done by coating MSD plates with TIM-3-Fc (R&D Systems) or TIM-3 his-tag (SinoBio) in Dulbecco's phosphate buffered saline (DPBS; Gibco). Plates were incubated overnight at 4°C, washed 3× with PBST buffer (0.1% Triton-X 100, Sigma), blocked with 5% Probumin (Millipore) in DPBS and stored at 4°C for 2 days. After washing, SULFO-TAG antibodies and galectin-9 were added to plates at serial dilutions of 1:10 in 25 μl/well diluent (2% Probumin/0.1% Tween20, Alfa Aesar/0.1% Triton-x 100/10% Stabilguard, SurModics/PBS, Gibco) for 1 hour at room temperature on a shaker. Plates were washed, Read Buffer T (Mesoscale) was added to each well, and plates were read on a MSD MESO Scale Discovery Sector Imager 6000, Model 1200 Plate reader. On the day of the MSD competition assay, a 96-well MSD plate was coated with TIM-3 Fc at 2 μg/ml in DPBS, 25-30 μl/well. The plate was incubated for 6 hours at room temperature (RT), washed, and blocked with 200 μl/well 5% Probumin in DPBS overnight at 4°C. After washing, sabatolimab was added in serial dilutions of 1:2. The plate was incubated for 1 hour at RT on a shaker, washed, and 3 μg/ml of competing human galectin-9 SULFO-TAG was added. The plate was incubated for 1 hour at RT on a shaker, washed, and Read Buffer T was added to the plate before reading on a MSD MESO Scale Discovery Sector Imager 6000, Model 1200 Plate reader.

#### Galectin-9 competition Luminex assay

Recombinant human galectin-9 (R&D Systems) was conjugated to PE using the Lightning Link R-PE Antibody Labeling Kit (novusbio) and TIM-3 Fc (R&D Systems) was coupled to microspheres using Luminex xMAP Antibody Coupling Kit (Luminex). On the day of the competition assay, 60 μl of 5 × 10^6^ 2 μg/ml Luminex TIM-3 Fc beads were diluted in 2.94 ml Assay Buffer (Millipore). 25 μl/well of diluted beads were added to a non-binding mylar 96-well plate (Grienier). 25 μl/well of antibodies were serially diluted in Assay Buffer. Plates were incubated for 1.5 hours at RT on a shaker, washed, and human galectin-9 PE at a concentration of 4 μg/ml was added. Plates were incubated for 45 minutes at RT on a shaker, washed, and 9 μl/well of Sheath Fluid (Luminex) was added to the wells. Plates were read on a FlexMap 3D.

#### TIM-3/MBG220 complex generation

Human TIM-3 was co-expressed with MBG220 Fab in Expi293^®^ cells to produce complex for crystallography. In detail, 0.3 mg of plasmid encoding TIM-3 was mixed with 0.15 mg of plasmid encoding heavy chain of MBG220 Fab and 0.15 mg of plasmid encoding light chain of MBG220 Fab, diluted into 30 ml of Opti-MEM^®^ I medium (Life Technologies), and incubated with 1.5 mg of Polyethylenimine (Polysciences) in 30 ml of the same medium for 30 minutes. The mixture was then added into 0.6L of Expi293^®^ cells growing in suspension in Expi293^®^ Expression medium (Life Technologies) at 2 × 10^6^ cells/ml at 37°C with 8% of CO_2_ for transfection. After 5 days, the medium containing TIM-3/MBG220 Fab complex was harvested by centrifugation and 5 ml of Ni-NTA resin was added into the medium while stirring at 4°C overnight. The next day, beads were packed into a gravity column and washed with 25 mM Hepes pH 7.4, 150 mM NaCl (Hepes buffered saline [HBS]) supplemented with 20 mM of imidazole. The complex was eluted with 3 column volumes of HBS with 500 mM of imidazole, and then dialyzed in HBS at 4°C. After 24 hours, the complex was incubated with 1/10 (w/w) of PNGaseF (purified in-house) at 37°C overnight to remove N-linked glycosylation, and then bound back to 5 ml of Ni-NTA resin, washed with HBS to remove PNGaseF and eluted with HBS plus 500 mM of imidazole. The eluate was concentrated and loaded onto HiLoad 16/600 Superdex 75 PG (GE Healthcare) size exclusion column equilibrated in HBS, and peak fractions containing purified TIM-3/MBG220 Fab complex were analyzed by SDS-PAGE, pooled, and concentrated for crystallization. Crystals for data collection were grown by hanging drop vapor diffusion at 20°C using 0.1 μl of the TIM-3/MBG220 Fab complex mixed with 0.1 µl of reservoir solution containing 0.04M potassium phosphate monobasic, 16% (w/v) PEG 8000, and 20% (v/v) Glycerol. Crystals were flash cooled in liquid nitrogen before data collection.

#### X-ray crystallography studies

Diffraction data were collected at beamline 17-ID at the Advanced Photon Source (Argonne National Laboratory, USA), and processed using Autoproc (version 1.1.5, Global Phasing, LTD). The data of TIM-3/MBG220 Fab were processed to 2.0 Å in space group P21 with cell dimensions a= 84.3 Å, b= 93.0 Å, c= 85.3 Å, α= 90°, β= 114°, and γ = 90°. The structure of the complex was solved by molecular replacement using Phaser (version 2.5.5) [33] with structures of mouse TIM-3 (PDB ID: 3KAA) and a Fab (in-house structure) as search models. The final model was built in COOT (version 0.6 pre) [34] and refined using Phenix (version 1.9) [35]. The Rwork and Rfree values were 17.5% and 22.1%, respectively; and the root-mean-square (r.m.s) deviation values of bond lengths and bond angles are 0.007Å and 1.1°, respectively. Epitope was defined as residues of TIM-3 that contain atoms within 5Å to any atom in MBG220 Fab, identified by CONTACT in CCP4 program suite (version 6.2.0) [36].

#### T-cell-mediated killing by flow cytometry

THP-1/TIM-3-Flag cells (TIM-3 over-expressers) were labeled with 2 μM CFSE (Carboxyfluorescein succinimidyl ester, Thermo Fisher Scientific), and THP-1 parental cells were labeled with 2 μM CTV (Thermo Fisher Scientific), according to the manufacturer’s instructions. THP-1 cells were mixed at a 1:1 ratio for a total of 100,000 THP-1 cells per well (50,000 THP-1 TIM-3-Flag and 50,000 THP-1 parental cells) and co-cultured for 3 days with 100,000 T cells purified using a human pan T-cell isolation kit (Miltenyi Biotec) from healthy human donor peripheral blood mononuclear cells (PBMCs) (Bioreclamation), in the presence of varying amounts of anti-CD3/anti-CD28 T-cell activation beads (Thermo Fisher Scientific) and 25 μg/ml sabatolimab whole antibody, sabatolimab Fab, sabatolimab Fab’2 or hIgG4 isotype control. On day 3 (~72 hours) of co-culture, cells were detected and counted by flow cytometric analysis. Differences in the killing of THP-1/TIM-3-Flag cells and THP-1 parental cells were determined by calculating the ratio of remaining cells at the end of the experiment.

#### T-cell-mediated killing by Incucyte

Human PBMCs were isolated from healthy donor whole blood (Medcor) by centrifugation of sodium citrate CPT tubes at 1800 × *g* for 20 minutes and were cultured with CellTracker™ Deep Red Dye-labeled (ThermoFisher) HNT-34 cells. Effector:target (E:T) ratios of 1:1, 1:2, and 1:3, with the target cell number constant at 10 000 cells/well, were tested. 100 ng/ml anti-CD3 (eBioscience) were added to the media, together with hIgG4 isotype control or sabatolimab at 1 μg/ml. The plate was placed in an Incucyte S3, and image phase and red fluorescent channels were captured every 4 hours for 4 days. At the completion of the assay, the target cell number (red events) was normalized to the first imaging time point using the Incucyte S3 2019B software. Killing was calculated as the difference between the signal for the condition analyzed and the isotype treated control, for each of the time points collected.

#### Immature monocyte-derived dendritic cell stimulation assay

Peripheral monocytes were isolated from PBMCs using untouched pan monocyte isolation kit (Miltenyi Biotec) and were differentiated into immature DCs over 6–7 days with a cocktail of recombinant human GM-CSF and IL-4, both at 100 ng/ml concentration. After 6–7 days, immature monocyte-derived DCs were collected by gentle pipetting and plated at 200 000–250,000 cells per well of a 96-well flat bottom plate in 100 μl of complete medium. LPS, R848 at a final concentration of 1 μg/ml, and sabatolimab or human IgG4 at a final concentration of 25 μg/ml were added, and cytokine release in the supernatant was measured 72 hours later using MSD assay, according to the manufacturer’s instructions.

#### Human fresh DC cultures

Human blood pan-DCs were purified from PBMCs (isolated from healthy human donor fresh blood from Medcor Cambridge internal donor program, or Bioreclamation) with a pan-DC enrichment kit (Miltenyi Biotec). Cells were plated in 96-well round bottom plates at 45 000–50 000 cells per well, in the presence of 25 μg/ml sabatolimab antibody or hIgG4 isotype control, for 16–24 hours. Cells were stimulated by adding 1 μg/ml LPS for 6 hours, with GolgiStop (BD Biosciences) added for the last 4 hours. Cells were then stained with antibodies for surface markers and intracellular cytokines, and analyzed by flow cytometry. cDC2 subtype cells (marked as HLADR^+^ CD11c^+^ CD1c^+^ cells) made up the majority of cells and showed the most robust results, and hence were focused on in the analysis.

#### ADCP assay using THP-1 and Raji cells

THP-1 cells were differentiated into a macrophage-like phenotype by adding 66 ng/ml of phorbal 12-myristate 13-acetate (PMA) to 1.5 × 10^7^ cells, and incubating the cells for 72 hours at 37°C with 5% CO_2_. 5 × 10^4^ cells (100 μl) of PMA-stimulated THP-1 cells were plated in a tissue culture-treated 96-well round bottom plate together with various concentrations of sabatolimab (or hIgG4 isotype control) for 10 minutes at RT prior to the addition of target cells. In some experiments, Fc-blocking antibodies (Fc block, 10 μg/ml) or Latrunculin A (1 μg/ml) were added to THP-1 cells together with sabatolimab or hIgG4, and incubated for 10 minutes at RT. After 10 minutes, 2.5 × 10^5^ CFSE-labeled Raji cells engineered to express human TIM-3 (hTIM-3 o.e. cells, 100 μl) were added to each well, and the plate was incubated for 1 hour at 37°C at 5% CO_2_. After 1 hour, cells were washed with PBS, stained for CD11c, and run on a BD LSR Fortessa flow cytometry machine. Data were analyzed using FlowJo analysis software and graphed using GraphPad Prism. Antibody-mediated cell phagocytosis (ADCP) activity was determined as percentage of CFSE^+^CD11c^+^ events.

#### ADCP assay using PBMCs and leukemia cells

Human PBMCs were separated from whole blood (from the internal donor program at Novartis) using Leucosep tubes, and monocytes were isolated following the Miltenyi Biotech Classical Monocyte Isolation Kit. Cells were plated in a 96-well round bottom plate at a concentration of 5 × 10^4^ cells/well and were incubated for 7 days with 50 ng/ml M-CSF at 37°C. On day 7, various concentrations of sabatolimab (or hIgG4) were added to the cells for 10 minutes at RT, followed by addition of 5 × 10^4^ cells (100 μl) of CFSE-labeled target cells (SKM-1 or HNT-34). Plates were incubated for 4 hours at 37°C at 5% CO_2_. After 4 hours, cells were washed with PBS, stained for CD11c, and run on a BD LSR Fortessa flow cytometry machine. Data were analyzed using FlowJo analysis software and graphed using GraphPad Prism. ADCP activity was determined as percentage of CFSE^+^CD11c^+^ events.

#### Fc-reporter assay

Raji cells engineered to express TIM-3 were co-cultured with Jurkat cells stably transfected to overexpress FcγRIa (CD64) and a luciferase reporter gene under the control of an NFAT (nuclear factor of activated T cells) response element. Graded concentrations (500 ng/ml to 6 pg/ml) of sabatolimab were added, and Fc-engagement was readout as luciferase signal.

## Results

### Generation and blocking properties of sabatolimab

**Figure 1.**
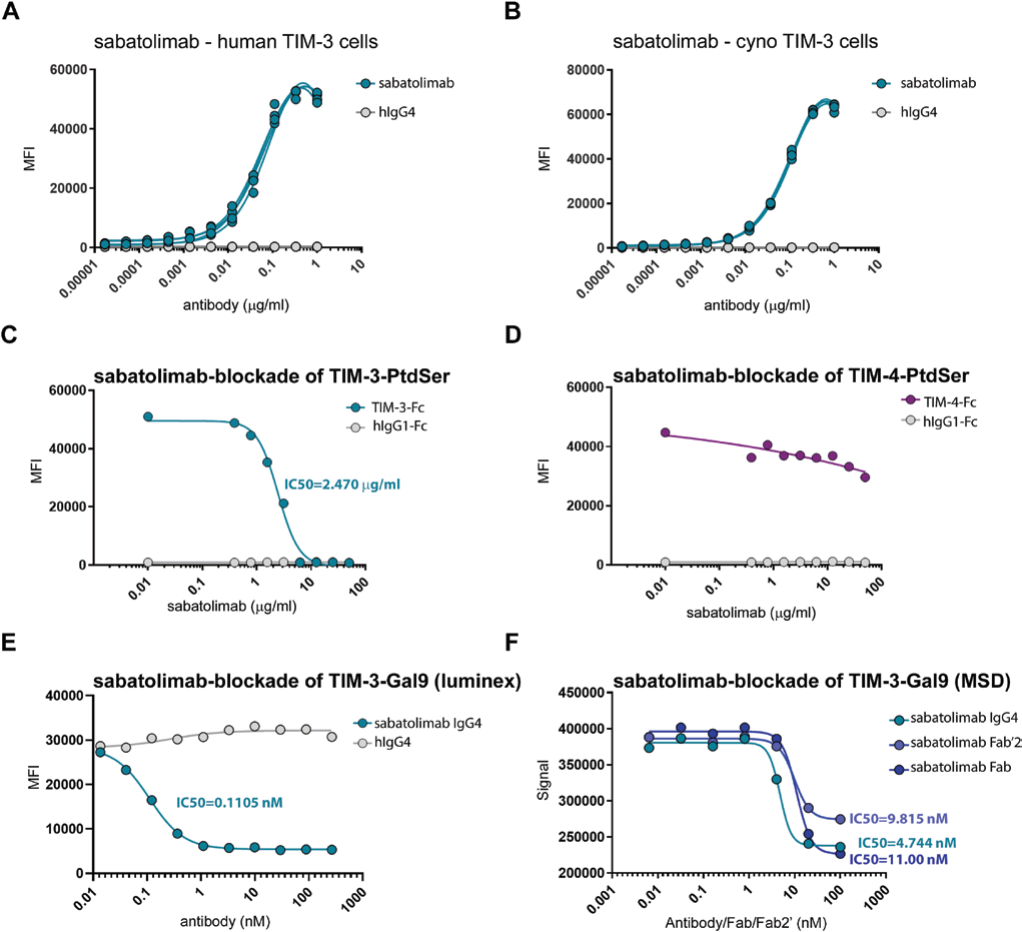
Blockade of TIM-3 ligand binding by sabatolimab. (A,B) Binding of sabatolimab to human and cyno TIM-3, as assessed by flow cytometric analysis on 300.19 cells engineered to express human or cyno TIM-3. (C) PtdSer exposure in the outer cell membrane leaflet was induced by staurosporine-mediated induction of apoptosis in human U937 monocytes and the ability of sabatolimab to block TIM-3 interaction with PtdSer was measured using flow cytometric analysis. Sabatolimab blocked the TIM-3-PtdSer interactions in a dose dependent manner. (D) In the same assay, sabatolimab did not block TIM-4:PtdSer interactions. (E,F) Sabatolimab showed a dose-dependent inhibition of TIM-3-galectin-9 interactions, as measured by luminex assay (E) or MSD (F).

Sabatolimab belongs to the IgG4/κ isotype subclass and contains a serine to proline substitution in the hinge region of the heavy chain (S228P) to reduce Fab arm exchange. Sabatolimab was initially developed by immunizing TIM-3-deficient mice with human TIM-3-Ig fusion protein and using hybridoma technology. The murine lead candidate was then humanized, and sabatolimab clone was selected. Sabatolimab binds human recombinant TIM-3, with a *K*_d_ of 0.167 ± 0.008 nM (Table 1; Supplementary Fig. 1A). Binding of sabatolimab to murine TIM-3 was undetectable, demonstrating that sabatolimab is not rodent cross-reactive (Supplementary Fig. 1B). Further flow-cytometry based tests using 300.19 cells engineered to express either the human TIM-3 or the cynomolgus monkey TIM-3 demonstrated binding of sabatolimab to both human and cyno TIM-3 (Fig. 1A and B). In these conditions, the *K*_d_ values for sabatolimab were determined to be 0.5 ± 0.1 nM on human TIM-3, and 0.9 ± 0.1 nM on cynomolgus monkey TIM-3 (Table 2).

**Table 1.** Biacore binding of sabatolimab to recombinant human TIM-3/His

		*K*_A_ (1/Ms)	*K*_D_ (1/s)	*K*_D_ (M)
Human	Sample 1	7.28E+05	1.28E−04	1.76E−10
	Sample 2	7.83E+05	1.27E−04	1.62E−10
	Sample 3	7.62E+05	1.24E−04	1.63E−10
	Average	7.58E+05	1.26E−04	1.67E−10

**Table 2.** Binding of sabatolimab to cells expressing human/cyno TIM-3 (flow cytometry)

		K_D_ (μg/ml)	Mean	SD
Human	Sample 1	0.06657	0.07641	0.019458
	Sample 2	0.1039		
	Sample 3	0.0596		
	Sample 4	0.07557		
Cyno	Sample 1	0.1217	0.13675	0.018939
	Sample 2	0.1192		
	Sample 3	0.1553		
	Sample 4	0.1508		

Sabatolimab was tested for its ability to block the binding of human TIM-3-Ig to PtdSer, one of the four ligands for TIM-3 [3]. PtdSer exposure in the outer cell membrane leaflet was induced by staurosporine-mediated induction of apoptosis in human U937 monocytes [37], and the *K*_d_ for binding of TIM-3 and related TIM family member TIM-4 to PtdSer on apoptotic U937 cells was determined using serial dilutions of human TIM-3-Ig and human TIM-4-Ig fusion proteins [3]. As shown in Supplementary Fig. 1C, both TIM-3-Ig and TIM-4-Ig bound to PtdSer, with a *K*_d_ of 2.4 μg/ml and 5.4 μg/ml, respectively; therefore, 5 μg/ml was chosen as constant concentration for both fusion proteins for the blocking assay. Sabatolimab was then added to the assay, and blocking was detected by flow cytometric analysis. Under these circumstances, sabatolimab was effective in blocking the binding of PtdSer to TIM-3 (Fig. 1C), without affecting the binding of PtdSer to TIM-4 (Fig. 1D), demonstrating specificity.

Sabatolimab was also tested for the ability to block binding of another TIM-3 ligand, galectin-9, to its receptor, using a Luminex assay. To this end, PE-labeled galectin-9 was titrated and tested for binding to Luminex Magnetic beads coated with two different concentrations of TIM-3-Fc (Supplementary Fig. 1D). A fixed concentration of PE-labeled galectin-9 was then added to the TIM-3-Fc beads, together with increasing concentrations of sabatolimab, or isotype control. As shown in Fig. 1E, sabatolimab blocked the binding of TIM-3 to galectin-9, in a dose-dependent manner. These data were further confirmed using MesoScale discovery platform and galectin-9 Sulfo-Tag as substrate. Sabatolimab was also able to block galectin-9 binding to plate-bound TIM-3 in both the full IgG4 format as well as in Fab fragments, as shown in Fig. 1F.

Altogether, these data sets demonstrate that sabatolimab inhibits the binding of PtdSer and galectin-9 to TIM-3, supporting its use to prevent engagement of the TIM-3 receptor.

### Crystal structure of TIM-3 antibodies

**Figure 2.**
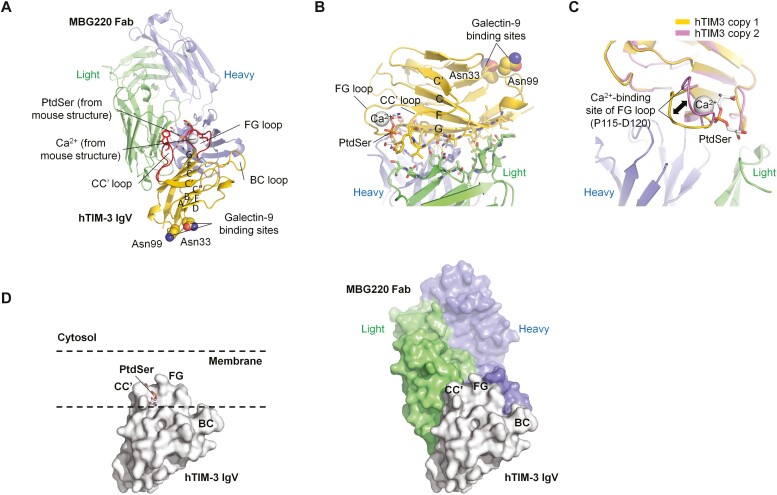
Structure of MBG220 Fab binding to TIM-3. (A) Overall structure of MBG220 Fab binding to TIM-3. MBG220 binds to the GFCC’ side of TIM-3 IgV domain, close to the CC’ and FG loop responsible for ligand binding. PtdSer and Ca^2+^ ion are modeled from the mouse TIM-3 structure to indicate ligand binding pocket. The residues corresponding to the proposed galectin-9 binding in mouse TIM-3 (Asn33 and Asn99) are also labeled. (B) Close-up view of the MBG220-TIM-3 interface. The paratope and epitope residues are shown as sticks. (C) Close-up view of the Ca^2+^ binding loop of TIM-3. (D) Comparison of PtdSer-mediated membrane penetration of mouse TIM-3 (left panel) and binding of MBG220 to human TIM-3 (right panel). The two TIM-3 structures are oriented the same way.

The structural features of TIM-3 antibodies were derived by hydrogen-deuterium exchange mass spectrometry epitope mapping studies and crystallography of human TIM-3 (IgV domain) bound to the Fab fragment of MBG220, one of the humanized antibodies derived from the parental clone from which sabatolimab was selected (Fig. 2 and Table 3). Notably, MBG220 differs by only one amino acid in the heavy chain CDR2 from the other humanized TIM-3 antibodies, in a location that is far distal (> 6Å) to the epitope and thus would not change antigen binding. Therefore, the crystal structure results described herein for MBG220 are also applicable to sabatolimab. Briefly, human TIM-3 was co-expressed with MBG220 Fab in mammalian cells to produce the purified complex, and protein crystallography was employed to generate atomic resolution data for TIM-3 bound to MBG220 Fab to define the epitope (Fig. 2A). The interaction surface on TIM-3 was determined to be formed by several continuous and discontinuous (i.e. noncontiguous) sequences in the CC’ and FG loops of the IgV domain of TIM-3, mapping the epitope proximal to the preserved TIM family PtdSer binding cleft [3], consistent with the observed disruption of PtdSer binding to TIM-3 by sabatolimab. A close-up view of the epitope is shown in Fig. 2B, highlighting that MBG220 is mainly engaging the G and F β-strands and part of the PtdSer-binding CC’ loop.

**Table 3.** Statistics of X-ray crystal structure determination for MBG220/TIM-3 complex

Data collection Statistics (17-ID, APS)	
Space group	P2_1_
a, b, c (Å)	84.3, 93.0, 85.3
a, b, c (Å)	90, 114, 90
Wavelength (Å)	1.0
Resolution range (Å)	77 - 2.0
Completeness (%)	98.1 (99.0)
R_merge_ (%)	0.100 (0.519)
<I>/σ	9.1 (2.3)
Redundancy	3.3 (3.2)
Wison B factor (Å^2^)	25.8
**Refinement Statistics**	
Reflections	
Working Set	76760
Test set	3811
Number of atoms	9319
Rmsd Bonds (Å)	0.007
Rmsd angles (°)	1.1
R_work_ (%)	17.5
R_free_ (%)	22.1
B factor (Å^2^)	13.1
Ramachandran plot	
Most favored (%)	97.2
Additionally allowed (%)	2.7
Disallowed (%)	0.1

MBG220 did not directly contact the Ca^2+^-binding loop of TIM-3, and this loop showed different conformations in the two copies of TIM-3 in the asymmetric unit, indicating its flexibility (Fig. 2C). The FG loop and CC’ loop of TIM-3 form a pocket (so called metal ion-dependent ligand-binding site, MILIBS) that has been shown by crystal structure to bind Ca^2+^ and PtdSer simultaneously (Fig. 2D, left) [3]. This binding is thought to help TIM-3-expressing cells engage and penetrate the membrane of apoptotic cells (which display PtdSer) for engulfment. The crystal structure of TIM-3/MBG220 Fab indicated that MBG220 binds the PtdSer-binding loops of the human TIM-3 IgV domain, and the attacking angle of the antibody supports the blockade of PtdSer-mediated membrane penetration of TIM-3 (Fig. 2D, right).

Interestingly, structural comparison between human and mouse TIM-3 (BALB/c) IgV domains revealed striking differences between the two molecules (Supplementary Fig. 2A) [38]. Part of the proposed galectin-9 binding site in mouse TIM-3 (Asn74 and Asn90), for example, was not conserved in human TIM-3 (Supplementary Fig. 2B) and while the Ca^2+^ binding residues were conserved between human and mouse TIM-3, the loop was in a slightly different conformation in human TIM-3, probably due to absence of Ca^2+^ ion (Supplementary Fig. 2C). The residues binding to the carboxyl group of PtdSer in mouse TIM-3 were also not conserved in human TIM-3 (Ser61 and Gln62 become Glu62 at this loop position, Supplementary Fig. 2D), as they were not the CC’ loop residues binding to the hydrophobic moiety of PtdSer (Trp 60 in mouse becomes Val60 and Phe61 in human at this loop position, Supplementary Fig. 2E). The corresponding FG loop residues were conserved, but adopted a slightly different conformation in human TIM-3, likely due to the absence of PtdSer. These key differences are consistent with the lack of binding of sabatolimab to murine TIM-3.

### Sabatolimab augments immune cell-mediated killing of TIM-3^+^ AML cells

**Figure 3.**
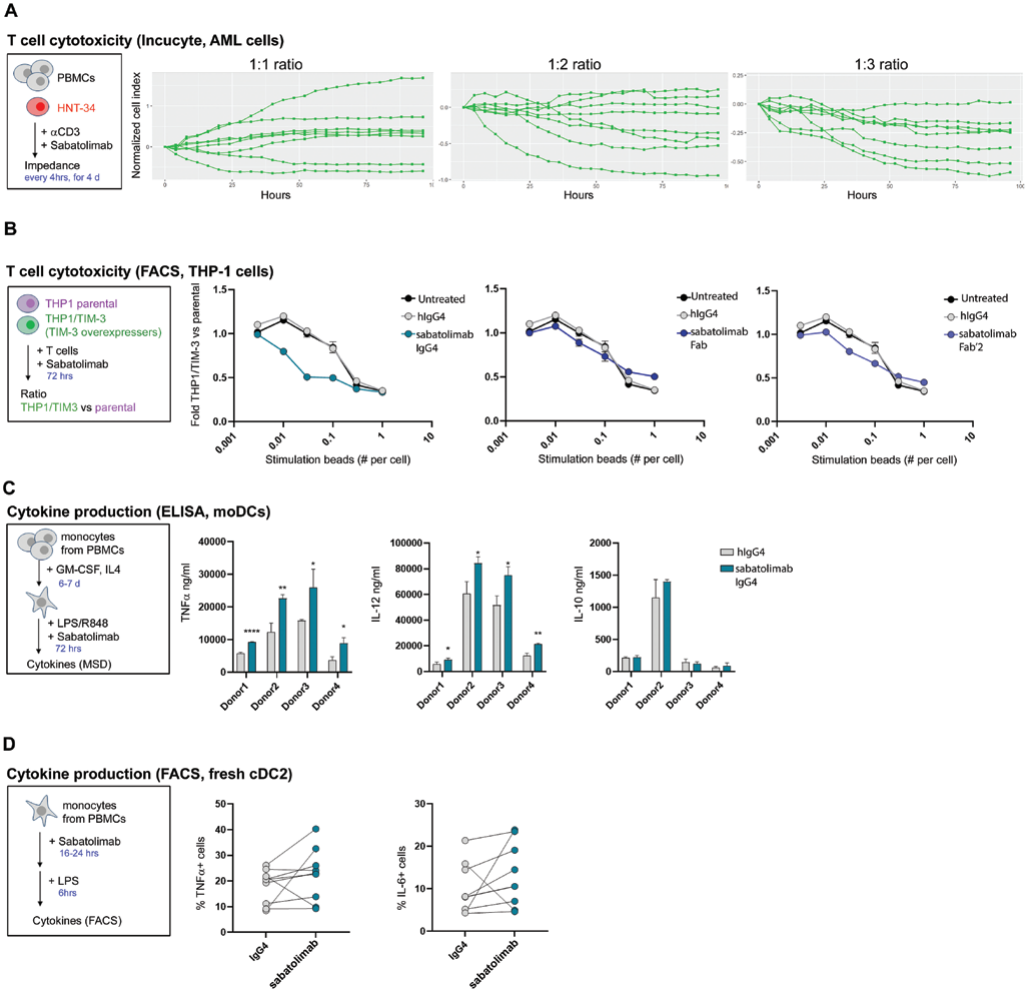
Immunomodulatory properties of sabatolimab *in vitro*. (A) HNT-34 AML cells were cultured with anti-CD3-activated PBMCs and cell viability was determined using Incucyte technology as change in cell impedance over the course of 4 days. Lines represent changes in the normalized cell index over time between sabatolimab- and hIgG4 isotype-treated samples at the highlighted ratios of HNT-34 target cells and PBMCs. Each line represents a sample from eight different donors (average from two replicates per sample per time point). (B) THP-1/TIM-3-Flag and THP-1 parental cells were labeled with two different dyes and cultured together with activated T cells (stimulated by the indicated amount of anti-CD3/CD28 beads as shown on the x-axis) in the presence of the indicated antibodies/antibody fragments. Graphs represent the ratio between the number of THP-1/TIM-3-Flag and THP-1 parental cells after co-culture. One representative of more than three experiments is shown. (C) Induction of IL-12, TNFα, and IL-10 by sabatolimab treatment in DCs generated from four independent donors as measured by ELISA. (D) Induction of TNFα and IL-6 by freshly isolated peripheral blood-derived DCs stimulated with LPS, as measured by flow cytometric analysis. Graph represents the percentage of cells positive for the analyzed cytokine in each treatment conditions. Each dot represents one donor; lines connect data in different conditions from the same donor; eight donors were analyzed. TNFα, *P* = 0.2939; IL-6, *P* = 0.3281.

TIM-3 has been associated with various immunological processes, prompting the analysis of the effects of sabatolimab on immune cell activity. Sabatolimab was first tested for its effect on immune-mediated target cell killing by culturing HNT-34 AML cells (expressing TIM-3, see also Supplementary Fig. 4B) with ratios of anti-CD3-activated PBMCs obtained from healthy donors. Cell viability was determined using Incucyte technology as change in cell impedance over the course of 4 days, and the effects of sabatolimab were calculated as the difference from co-cultures treated with hIgG4 isotype control. As shown in Fig. 3A, sabatolimab promoted PBMC-mediated cell killing of HNT-34 cells in certain donors, with an observable decrease in the normalized cell index. On the other hand, F36P cells, another AML cell line (secondary to MDS) that lacks surface TIM-3 expression, showed insensitivity to sabatolimab treatment when used in the same assay (data not shown). Taken together, these data suggest that sabatolimab-blockade of TIM-3 enhances immune-mediated killing of TIM-3^+^ leukemic cells *in vitro*, in a donor-dependent manner. Additional studies using primary T cells purified from healthy donor whole blood PBMCs were undertaken to further explore the activity of sabatolimab in the context of immune cell-mediated killing. Briefly, CFSE-labeled THP-1 AML cells engineered to stably overexpress TIM-3 (THP-1/TIM-3-Flag) and CTV-labeled THP-1 parental cells were co-cultured with purified T cells in the presence of anti-CD3/anti-CD28 T-cell activation beads and 25 μg/ml sabatolimab whole antibody, sabatolimab Fab or hIgG4 isotype control. Cells were then detected and counted by flow cytometry. The ratio between TIM-3-expressing THP-1 cells and parental THP-1 cells (‘fold’ in y-axis of graphs; Fig. 3B) was calculated and normalized to conditions without anti-CD3/anti-CD28 bead stimulation. As shown in Fig. 3B, expression of TIM-3 on target cells sensitized them to T-cell killing, a phenomenon that was further enhanced by addition of sabatolimab (but not sabatolimab antibody fragments), suggesting that the Fc-portion of sabatolimab may be important for sabatolimab-enhanced T-cell-mediated killing of TIM-3^+^ AML cells. The molecular mechanism at the bases of sabatolimab-mediated T-cell killing remains to be elucidated.

### Sabatolimab enhances cytokine secretion by myeloid cells

As TIM-3 has been associated with the modulation of cytokine production in myeloid cells [16], sabatolimab was also tested for its modulatory potential in DC cultures. Human immature DCs were generated from four healthy donors by culturing PBMCs with a cocktail of recombinant human GM-CSF and IL-4 over the course of 6–7 days, and then stimulated with LPS (TLR4 agonist) and R848 (TLR7/8 agonist). The production of IL12, TNFα, and IL10 was measured in cell culture supernatants after 72 hours of stimulation. As shown in Fig. 3C, TIM-3-blockade with sabatolimab significantly augmented secretion of IL-12 and TNFα in all four donors tested, while not affecting IL-10 levels. This finding was recapitulated using freshly isolated peripheral blood-derived DCs stimulated with LPS. cDC2 subtype cells (marked as HLADR^+^ CD11c^+^ CD1c^+^ cells) made up the majority of cells and showed the most robust results, and hence were focused on in the analysis. In these conditions, pre-treatment with sabatolimab increased the production of both TNFα and IL-6 in a donor-dependent manner, as measured by intracellular staining and flow cytometry (Fig. 3D).

Altogether, these data suggest that sabatolimab-blockade of TIM-3 following TLR-mediated stimulation of myeloid cells increases the activation state of the DCs, as seen by enhanced secretion of pro-inflammatory cytokines.

### ADCP is a feature of sabatolimab

**Figure 4.**
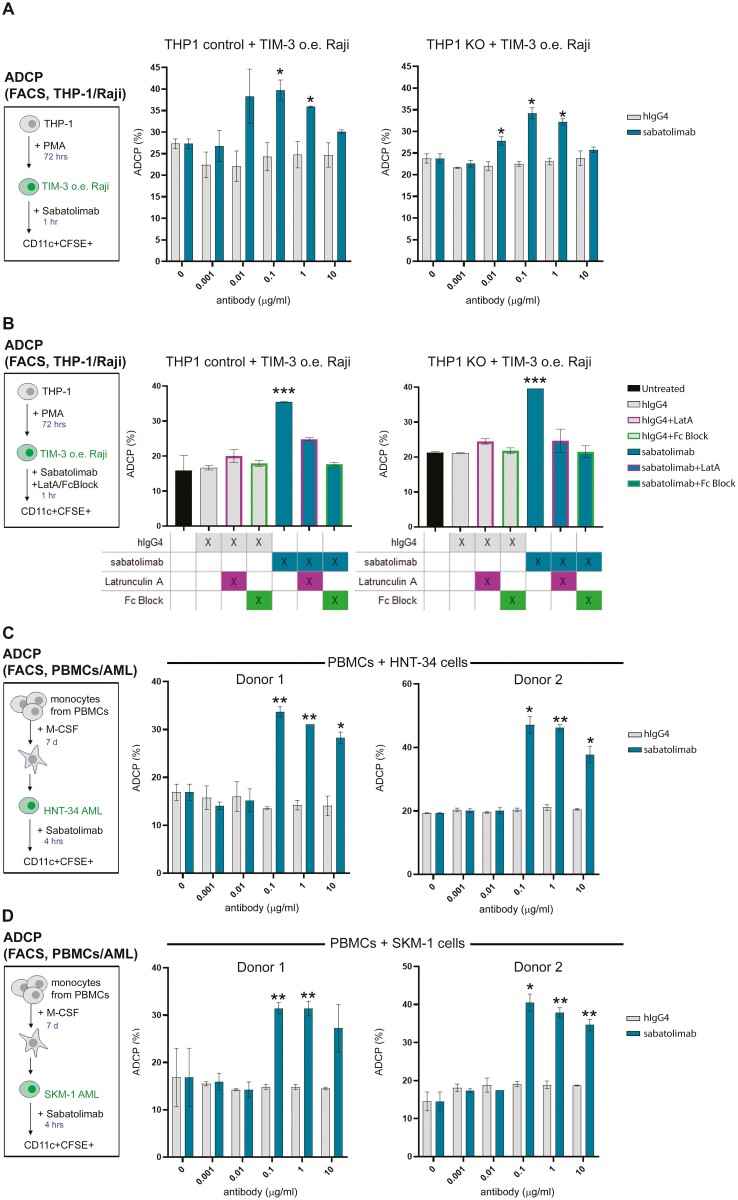
ADCP activity of sabatolimab *in vitro*. (A) THP-1 cells (TIM-3 sufficient, control, or TIM-3 deficient, KO) were cultured for 1 hour with Raji TIM-3 o.e. cells and treated with different concentrations of sabatolimab or hIgG4 isotype control in duplicates. ADCP was determined by flow cytometric analysis as % of CFSE^+^CD11c^+^ cells. Plots depict means ± SD for each condition. (B) THP-1 cells (TIM-3 sufficient, control, or TIM-3 deficient, KO) cultured as in A with with Raji hTIM-3 o.e. cells were treated with sabatolimab or hIgG4 isotype control, with Latrunculin A or Fc-blocking antibody added to some of the wells in duplicates. ADCP was determined by flow cytometric analysis as % of CFSE^+^CD11c^+^ cells. Plots depict means ± SD for each condition. (C,D) Macrophages differentiated from PBMC-isolated monocytes were cultured for 4 hours with CFSE-labeled leukemia cells (HNT-34, in C or SKM-1, in D) and treated with different concentrations of sabatolimab or hIgG4 isotype control in duplicates. ADCP was determined by flow cytometric analysis as % of CFSE^+^CD11c^+^ cells. Plots depict means ± SD for each condition. Two separate donors are shown for each cell line. Asterisks mark statistically significant *P* values between sabatolimab- and isotype control-treated samples (*T* test *P < 0.05, **P < 0.005, ***P < 0.001).

Finally, we evaluated the activity of sabatolimab in inducing ADCP of TIM-3^+^ target cells. THP-1 AML (effector) cells were differentiated into phagocytic cells by treatment with PMA, as determined by flow cytometric analysis to monitor the expression of CD11b, CD11c, and TIM-3 (Supplementary Fig. 3A). THP-1 Knock-Out (KO) and THP-1 Knock-Out Control cells were used to determine a requirement for TIM-3 on phagocytes with regard to ADCP activity. As target cells, Raji B lymphoma engineered to overexpress human TIM-3 (hTIM-3 o.e.) were used. Validation of the over-expressing Raji cells by flow cytometric analysis is shown in Supplementary Fig. 3B. PMA-stimulated THP-1 cells (KO and control) were co-cultured with Raji hTIM-3 o.e. cells, and sabatolimab was added to some wells at various concentrations. ADCP was determined by gating on CD11c^+^ cells (to identify THP-1 cells based on their upregulation of CD11c upon PMA stimulation) that were also positive for CFSE (representing phagocytosis of CFSE-labeled target cells, Supplementary Fig. 3C). As shown in Fig. 4A, the addition of sabatolimab, but not hIgG4 isotype control, enhanced the phagocytosis of Raji hTIM-3 o.e. cells by THP-1 control cells. Silencing of TIM-3 in THP-1 cells (TIM-3 KO) did not abrogate ADCP activity, suggesting that in this context, TIM-3 expression on phagocytes may be dispensable for sabatolimab-dependent phagocytosis of TIM-3^+^ target cells. Additionally, no ADCP activity was observed using Raji parental cells (with no TIM-3 expression), supporting that ADCP induced by sabatolimab depends on the expression of TIM-3 on target cells (data not shown).

As ADCP has been shown to require cytoskeletal remodeling and FcR-engagement, the activity of sabatolimab was tested in the THP-1-Raji ADCP assay together with Latrunculin A or Fc-blocking antibodies. As shown in Fig. 4B, Latrunculin A abolished the ADCP activity observed with sabatolimab in both the THP-1 control and KO cells, suggesting that sabatolimab-mediated ADCP requires intact cytoskeletal dynamic processes. The addition of Fc-blocking antibodies (Fc block) similarly abrogated sabatolimab-induced ADCP (Fig. 4B), supporting the requirement of Fc-receptors for sabatolimab-dependent induction of ADCP *in vitro*. Consistently, sabatolimab exhibited a dose–response engagement of FcγRIa in a cell reporter assay using Jurkat cells stably transfected to express FcγRIa and a luciferase reporter gene under the control of an NFAT response element (Supplementary Fig. 3D). This finding is consistent with the notion that FcγRIa receptor binds all human IgG subclasses except IgG2, although with different affinity[39], and further describes the Fc-engagement properties of sabatolimab. Altogether, these data support the conclusion that sabatolimab is capable of inducing cellular phagocytosis of TIM-3-expressing target cells *in vitro*, and this function requires Fc-receptors and cytoskeletal remodeling.

Given the expression of TIM-3 on leukemic stem cells and blasts, the ADCP activity of sabatolimab was further tested using PBMC-derived monocytes as effector cells and myeloid leukemic cells as target cells. For these experiments, monocytes isolated from PBMCs were differentiated into macrophage-like cells by culturing them for 7 days with M-CSF, prompting their differentiation into CD11c^+^ TIM-3^+^ macrophages (Supplementary Fig. 4A). M-CSF-differentiated macrophages were then co-cultured with TIM-3-expressing HNT-34 or SKM-1 leukemia cell lines (Supplementary Fig. 4B), and sabatolimab was added at various concentrations. ADCP was determined by gating on CD11c^+^ macrophages that were also positive for CFSE (representing phagocytosis of CFSE-labeled target leukemia cells, Supplementary Fig. 4C). As shown in Fig. 4C and D, addition of sabatolimab, and not hIgG4 isotype control, enhanced phagocytosis of both HNT-34 and SKM-1 cells by primary macrophages obtained from both donors analyzed. Taken together, these data demonstrate that sabatolimab can facilitate phagocytic uptake of TIM-3-expressing leukemia target cells *in vitro*, supporting the notion that ADCP is a feature of sabatolimab.

## Discussion

Sabatolimab is a high-affinity, humanized anti-TIM-3 IgG4/κ (S228P) monoclonal antibody currently in development for high-risk MDS and AML. Herein, we have characterized the functional features of sabatolimab, demonstrating its blocking activity and implications for immune cell modulation, in support of its use in cancer patients.

Sabatolimab is cynomolgus monkey cross-reactive, and was shown to block the binding of PtdSer to cell surface-expressed TIM-3, consistent with the putative epitope ascertained from the crystallography studies. Sabatolimab was also shown to block the binding of galectin-9. It is possible that although TIM-3 antibodies may not directly provide physical occlusion of the galectin-9 binding site, binding to the ligand may require structure dynamics changes that are affected by the presence of sabatolimab. This hypothesis is further supported by the proximity of the galectin-9 binding site of human TIM-3 closer to the PtdSer cleft, in comparison to that of the murine TIM-3 [40], suggesting that binding of sabatolimab to the cleft could induce a conformation change of TIM-3 that may negatively impact accessibility of the galectin-9 site.

Disruption of the galectin-9-TIM-3 interaction by sabatolimab is of particular interest because of its implications for myeloid cell malignancies. Plasma levels of galectin-9 have been shown to be significantly elevated in patients with acute leukemia transformed from MDS (AL-MDS) or with refractory anemia, in comparison to healthy controls, suggesting that galectin-9 production may be induced in MDS patients with advanced-stage disease, and that the TIM-3/galectin-9 axis may have a role in disease progression and leukemic transformation [28]. Mechanistically, TIM-3 is expressed on the surface of leukemic stem cells and blasts in AML, MDS, and CMML, and a TIM-3 autocrine feedback loop with its ligand galectin-9 has been reported to enhance LSC self-renewal [26]. By halting the TIM-3/galectin-9 interaction in leukemic cells, sabatolimab has therefore the potential to limit this autocrine feedback loop, and may have a direct impact on leukemic cells, limiting their self-renewal and reducing cancer growth.

Sabatolimab did show functional activity in several immunological *in vitro* assays with normal PBMCs, promoting pro-inflammatory cytokine secretion and T-cell-mediated killing of TIM-3^+^ cells. These data are in agreement with previous reports on the role of TIM-3 in immune cells, and highlight the immunomodulatory potential of sabatolimab. These findings are also consistent with emerging data demonstrating that conditional deletion of TIM-3 in DCs increases NLRP3 inflammasome activation in DCs, resulting in accumulation of IL-1β/IL-18 [16], therefore strengthening a function for TIM-3 in myeloid cells and supporting its broader role in the tumor microenvironment beyond that of checkpoint receptors in T cells. On top of these functions, sabatolimab was also shown to elicit ADCP of TIM-3^+^ leukemic cells *in vitro*, adding one more pillar of activity to the mechanistic paradigm for TIM-3 blockade. The individual mechanistic contributions to the eradication of TIM-3^+^ leukemic cells and blasts, as reflected in the promising clinical activity observed in a phase 1 trial of sabatolimab in patients with myeloid leukemias [41], remains a focus of future studies. However, the diverse effects of sabatolimab on different cellular compartments clearly highlight a striking distinction from canonical checkpoint blockades, including PD-1/PD-L1 targeting agents. Modeling these activities in preclinical *in vivo* settings has been challenged by the fact that sabatolimab is not murine cross-reactive, limiting its testing in immunocompetent animals. As data from the clinic continue to emerge, they will shed more light onto the activity of sabatolimab in patients with cancer and its global effects on the tumor microenvironment.

In conclusion, we herein reported the characterization of sabatolimab, a potent and specific monoclonal antibody against human TIM-3. Sabatolimab was shown to (i) enhance T-cell-mediated killing of TIM-3^+^ cells and promote inflammatory cytokine production by DCs; (ii) facilitate the phagocytic uptake of TIM-3-expressing target cells; and (iii) block the interaction between TIM-3 and its ligand galectin-9, potentially affecting the TIM-3/galectin-9 autocrine feedback loop in leukemic stem cells. Because of its broad mechanism of action, sabatolimab represents a novel immunotherapy with immuno-myeloid activity, holding promise for the treatment of myeloid cell neoplasms.

## Supplementary material

Supplementary data are available at *Immunotherapy Advances* online.

Supplementary Figure 1. TIM-3 ligand expression and titration. A. Representative biacore sensograms of sabatolimab binding to recombinant human TIM-3/His protein. B. Representative biacore sensograms of sabatolimab binding to recombinant murine TIM-3/His protein. C. TIM-3-Fc binding to phosphatidylserine on apoptotic cells is shown, with TIM-4-Fc and IgG1-Fc as control. D. PE labeled galectin-9 was titrated and tested for binding to Luminex Magnetic beads coated with two different concentrations of TIM-3 Fc. Galectin-9 PE was titrated and added to beads, showing binding to TIM-3 Fc at both bead concentrations in a dose-dependent manner.

Supplementary Figure 2: Structural comparison between human and mouse TIM-3 IgV domains. A. Structural overlay of mouse (colored in silver, PDB ID 3KAA) and human (colored in gold) TIM-3 IgV domains, areas of interest are boxed and labeled. B. Part of the proposed galectin-9 binding site in mouse TIM-3 (Asn74 and Asn90) and changes in human TIM-3. C. Ca^2+^ binding residues and loop conformation. D. Residues binding to the carboxyl group of PtdSer. E. CC’ loop residues binding to the hydrophobic moiety of PtdSer and changes in conformation in the FG loop.

Supplementary Figure 3. Tool validation and gating strategy for ADCP assay using THP-1 cells. A. Expression of CD11c, CD11b, and TIM-3 in THP-1 cells. Red histograms represent expression at steady state; blue histograms represent expression in PMA-stimulated cells. THP-1 KO, TIM-3 KO THP-1 cells; THP-1 control, TIM-3 sufficient THP-1 cells. B. WT Raji cells and Raji cells engineered to express human TIM-3 (Raji hTIM-3 o.e.) were stained with an APC-TIM-3 antibody, and expression of TIM-3 was determined by flow cytometry. A representative plot is shown. C. Representative gating strategy used to determine ADCP with CD11c stained THP-1 cells and CFSE-labeled TIM-3-expressing Raji cells (Raji hTIM-3 o.e.). D. Sabatolimab-dependent engagement of FcR was tested using Raji hTIM-3 o.e. cells as target cells in a co-culture assay with engineered effector Jurkat cells stably transfected to overexpress FcγRIa (CD64) and a luciferase reporter gene under the control of an NFAT response element. Each line indicates treatment with one of three different lots of sabatolimab.

Supplementary Figure 4. Tool validation and gating strategy for ADCP assay using primary human phagocytes. A. Expression of CD11c and TIM-3 in PBMC-derived monocytes after 7 days of differentiation with M-CSF. Red histograms represent expression of the indicated marker, blue histograms represent unstained control. Representative plots are shown. B. HNT-34 and SKM-1 leukemia cell lines were stained with a BV421-TIM-3 antibody, and expression of TIM-3 was determined by flow cytometry. Blue histograms represent expression of TIM-3, red histograms represent unstained control. Representative plots are shown. C. Representative gating strategy used to determine ADCP with CD11c stained primary phagocytes and CFSE-labeled AML cells (HNT-34).

ltac019_suppl_Supplementary_Figure_S1

ltac019_suppl_Supplementary_Figure_S2

ltac019_suppl_Supplementary_Figure_S3

ltac019_suppl_Supplementary_Figure_S4

## Data Availability

Data are available upon request.

## Abbreviations

ADCP: Antibody-mediated cell phagocytosis
DC: Dendritic cell
FBS: Fetal bovine serum
FITC: Fluorescein isothiocyanate
HMGB1: High-mobility group protein B1
KO: Knock-Out
LSC: Leukemic stem cell
MDS: Myelodysplastic syndrome
PBMC: Peripheral blood mononuclear cell
PE: Phycoerythrin
PMA: Phorbal 12-myristate 13-acetate
PtdSer: Phosphatidylserine
RT: Room temperature
TIM-3: T-cell immunoglobulin domain and mucin domain-3
