# Correction to: Characterization of sabatolimab, a novel immunotherapy with immuno-myeloid activity directed against TIM-3 receptor

**DOI:** 10.1093/immadv/ltad002

**Published:** 2023-02-18

This is a correction to: Stephanie Schwartz, Nidhi Patel, Tyler Longmire, Pushpa Jayaraman, Xiaomo Jiang, Hongbo Lu, Lisa Baker, Janelle Velez, Radha Ramesh, Anne-Sophie Wavreille, Melanie Verneret, Hong Fan, Tiancen Hu, Fangmin Xu, John Taraszka, Marc Pelletier, Joy Miyashiro, Mikael Rinne, Glenn Dranoff, Catherine Sabatos-Peyton, Viviana Cremasco, Characterization of sabatolimab, a novel immunotherapy with immuno-myeloid activity directed against TIM-3 receptor, *Immunotherapy Advances,* Volume 2, Issue 1, 2022, ltac019, https://doi.org/10.1093/immadv/ltac019

In the originally published version of this manuscript, the results from table 1 were incorrectly copied onto table 2

There seems to have been an issue with table 2, where the results have been accidentally copied over from table 1.

According to the author, table 2 should read:

**Table:** 

		K_D_ (μg/ml)	Mean	SD
Human	Sample 1	0.06657	0.07641	0.019458
	Sample 2	0.1039		
	Sample 3	0.0596		
	Sample 4	0.07557		
Cyno	Sample 1	0.1217	0.13675	0.018939
	Sample 2	0.1192		
	Sample 3	0.1553		
	Sample 4	0.1508		

This error has been corrected online.

