# Supplementary figures and images for: Characterization of sabatolimab, a novel immunotherapy with immuno-myeloid activity directed against TIM-3 receptor
Source: Immunother Adv. 2022 Aug 10;2(1):ltac019. doi: 10.1093/immadv/ltac019 (PMC9525012; doi:10.1093/immadv/ltac019)

Sabatolimab, Supplementary Figure 1

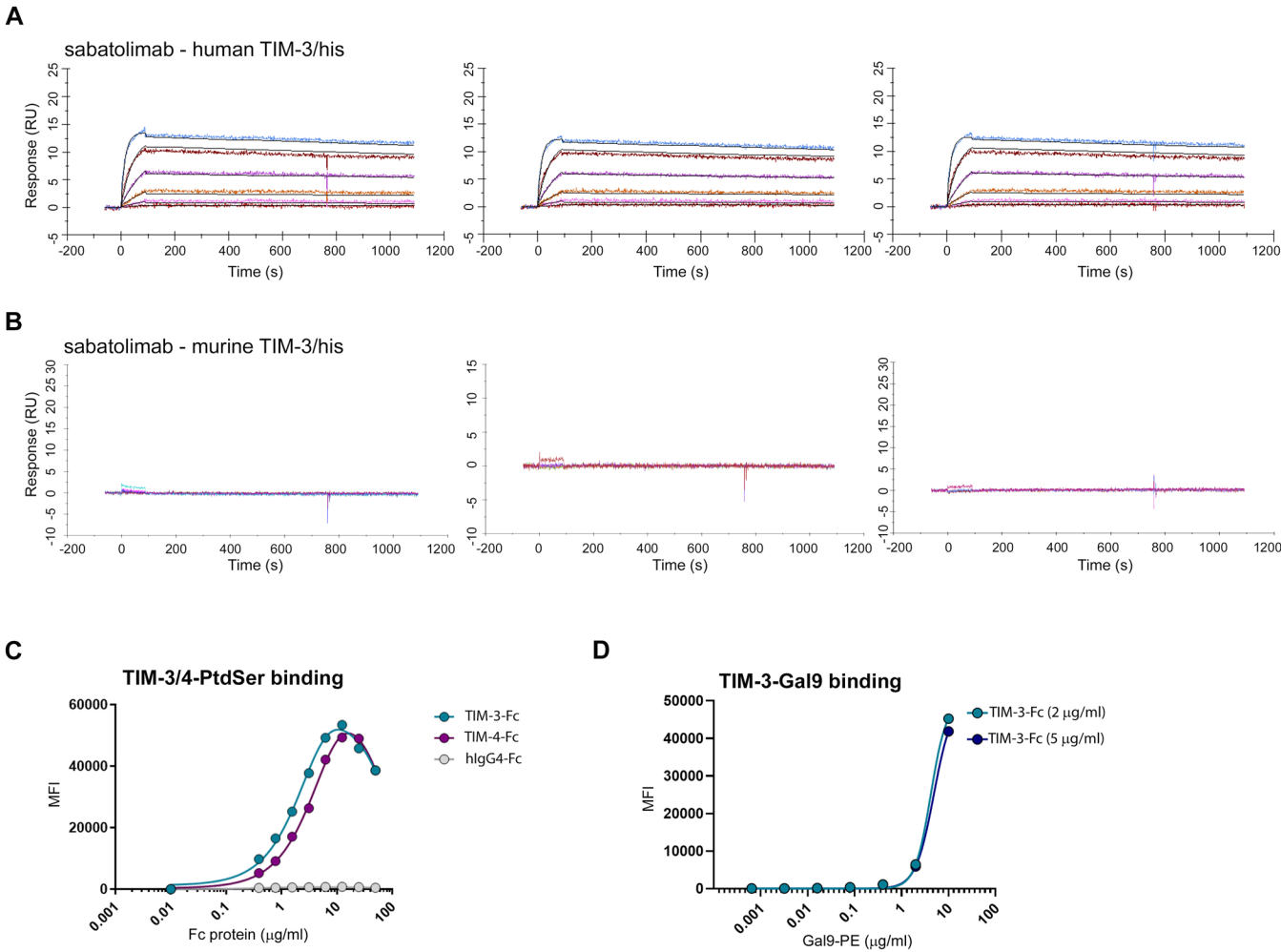

Supplement: ltac019_suppl_Supplementary_Figure_S1 [file ltac019_suppl_supplementary_figure_s1.pdf]

Sabatolimab, Supplementary Figure 2

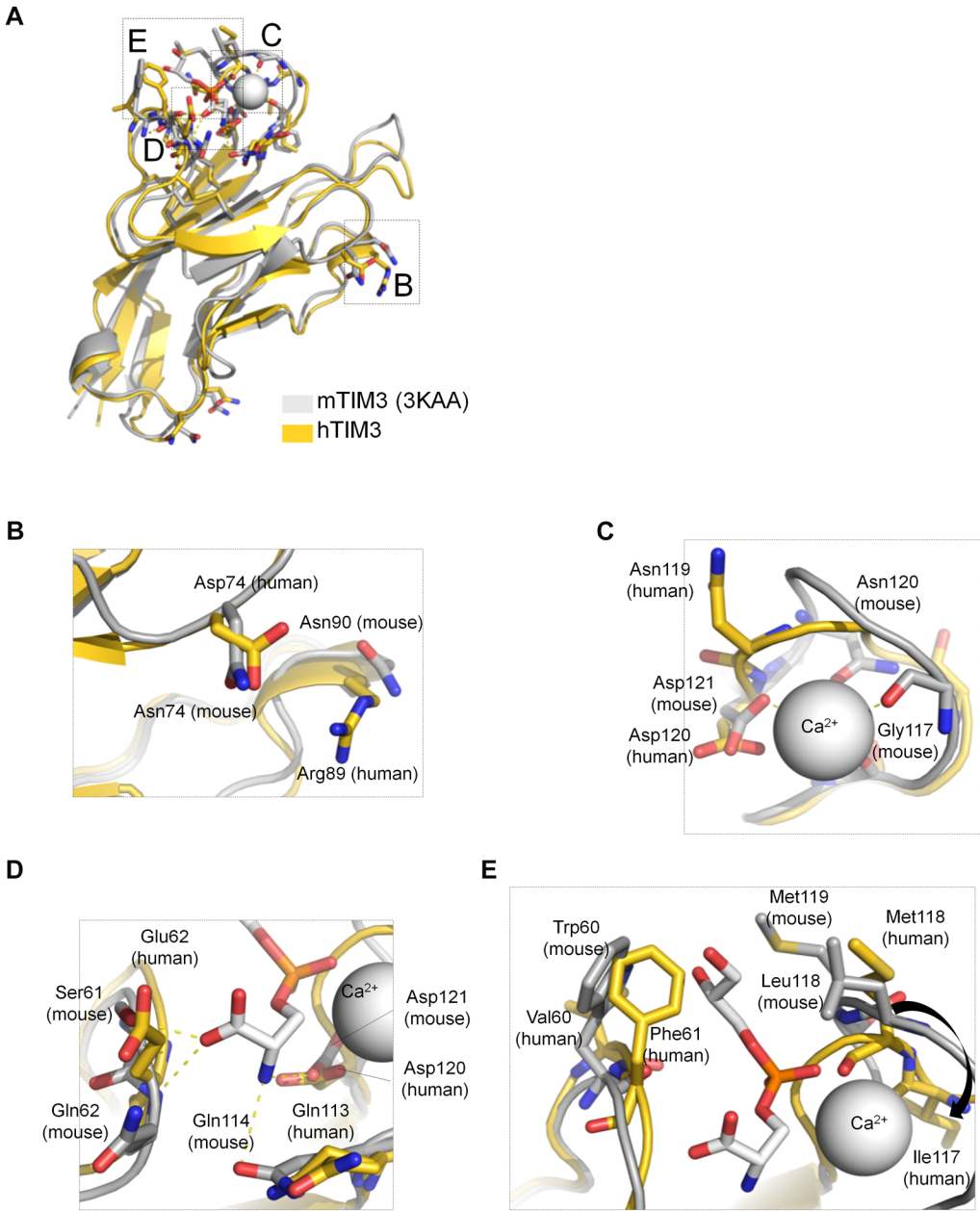

Supplement: ltac019_suppl_Supplementary_Figure_S2 [file ltac019_suppl_supplementary_figure_s2.pdf]

Sabatolimab, Supplementary Figure 3

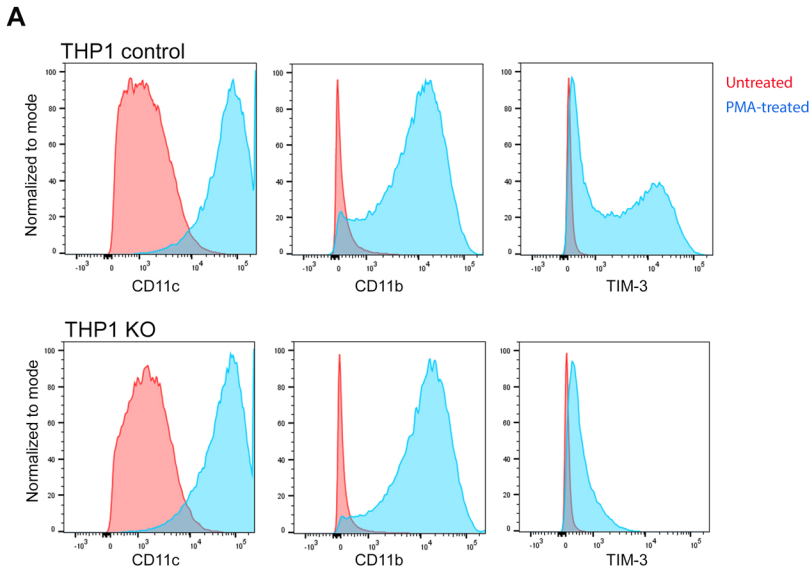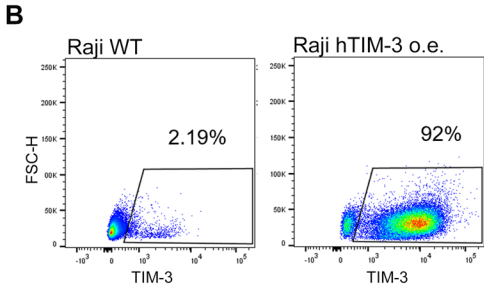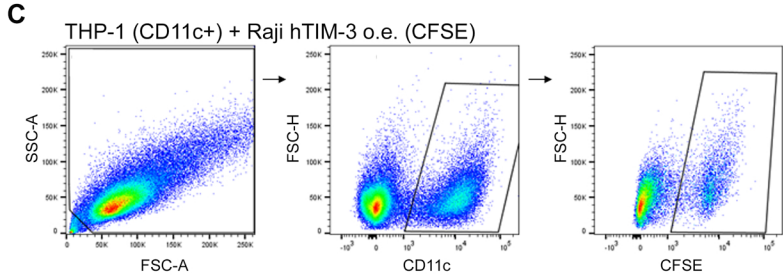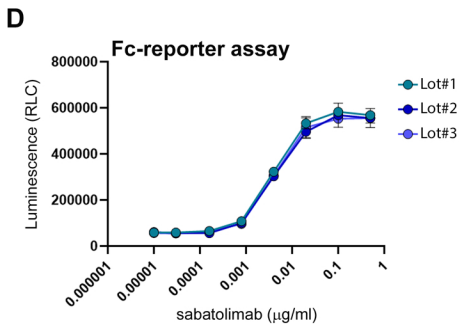

Supplement: ltac019_suppl_Supplementary_Figure_S3 [file ltac019_suppl_supplementary_figure_s3.pdf]

A

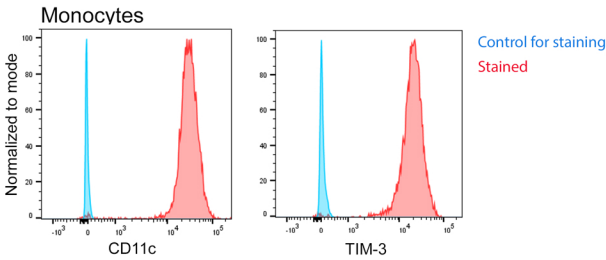

B

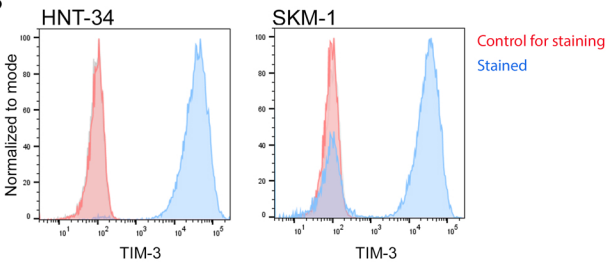

C

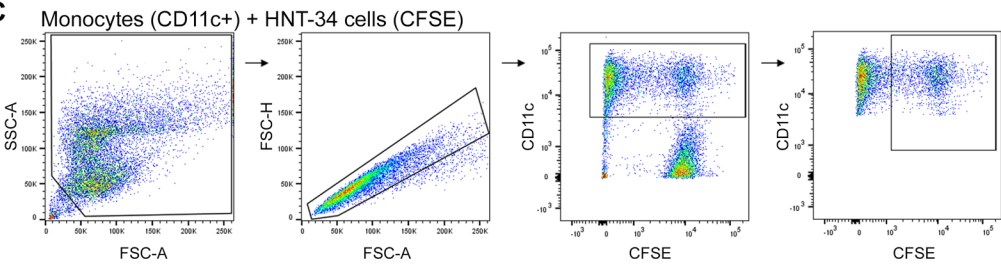

Supplement: ltac019_suppl_Supplementary_Figure_S4 [file ltac019_suppl_supplementary_figure_s4.pdf]
